# Importin α7 Is Essential for Zygotic Genome Activation and Early Mouse Development

**DOI:** 10.1371/journal.pone.0018310

**Published:** 2011-03-29

**Authors:** Franziska Rother, Tatiana Shmidt, Elena Popova, Alexander Krivokharchenko, Stefanie Hügel, Larissa Vilianovich, Michael Ridders, Katja Tenner, Natalia Alenina, Matthias Köhler, Enno Hartmann, Michael Bader

**Affiliations:** 1 Max-Delbrück-Center for Molecular Medicine, Berlin-Buch, Germany; 2 Center for Nephrology and Hypertension, Ostsee Clinic and Reha Clinic Damp, Damp, and Department of Nephrology and Hypertension, University of Kiel, Kiel, Germany; 3 Center for Structural and Cell Biology in Medicine, Institute for Biology, University of Lübeck, Lübeck, Germany; Ottawa Hospital Research Institute and University of Ottawa, Canada

## Abstract

Importin α is involved in the nuclear import of proteins. It also contributes to spindle assembly and nuclear membrane formation, however, the underlying mechanisms are poorly understood. Here, we studied the function of importin α7 by gene targeting in mice and show that it is essential for early embryonic development. Embryos lacking importin α7 display a reduced ability for the first cleavage and arrest completely at the two-cell stage. We show that the zygotic genome activation is severely disturbed in these embryos. Our findings indicate that importin α7 is a new member of the small group of maternal effect genes.

## Introduction

The importin α (also called karyopherin α) family comprises soluble transport factors that mediate the movement of proteins from cytoplasm to the nucleus in interphase cells [1]. Recent studies have extended the function of α-importins and shown that they are involved in spindle assembly and nuclear membrane formation in mitotic cells [2]–[4]. The precise mechanisms underlying these importin α functions have not been identified yet. During evolution, the family of importin α-genes was markedly expanded. While yeast expresses only one α importin, the invertebrates *D. melanogaster* and *C. elegans* have three paralogs and in mice, six different paralogs have been described [5]–[8]. In invertebrates the specific physiological role of importin α-paralogs has been studied, and distinct functions of single importin α genes in gametogenesis and early development have been revealed [9]–[11]. Functional characterizations of α importins in vertebrate development are scarce. We have recently shown that importin α5 (Kpna1, NM_008465.3) is not essential for brain development against expectations from studies in embryonic stem cells and a recent publication defines importin α2 (Kpna7, AY950703) as maternal effect protein [12].

Here, we studied the function of importin α7 (Kpna6, NM_008468.3) by gene targeting in mice and show that it is essential for early embryonic development. We show that importin α7 is a maternal protein present in oocytes. Oocytes lacking this protein can be fertilized but display a reduced ability for the first cleavage and a complete arrest at the two-cell embryo stage. Expression analyses for several marker genes of zygotic genome activation (ZGA) showed that ZGA is severely disturbed in these embryos.

## Results

### Importin α7 deficient mice are viable

**Figure 1:**
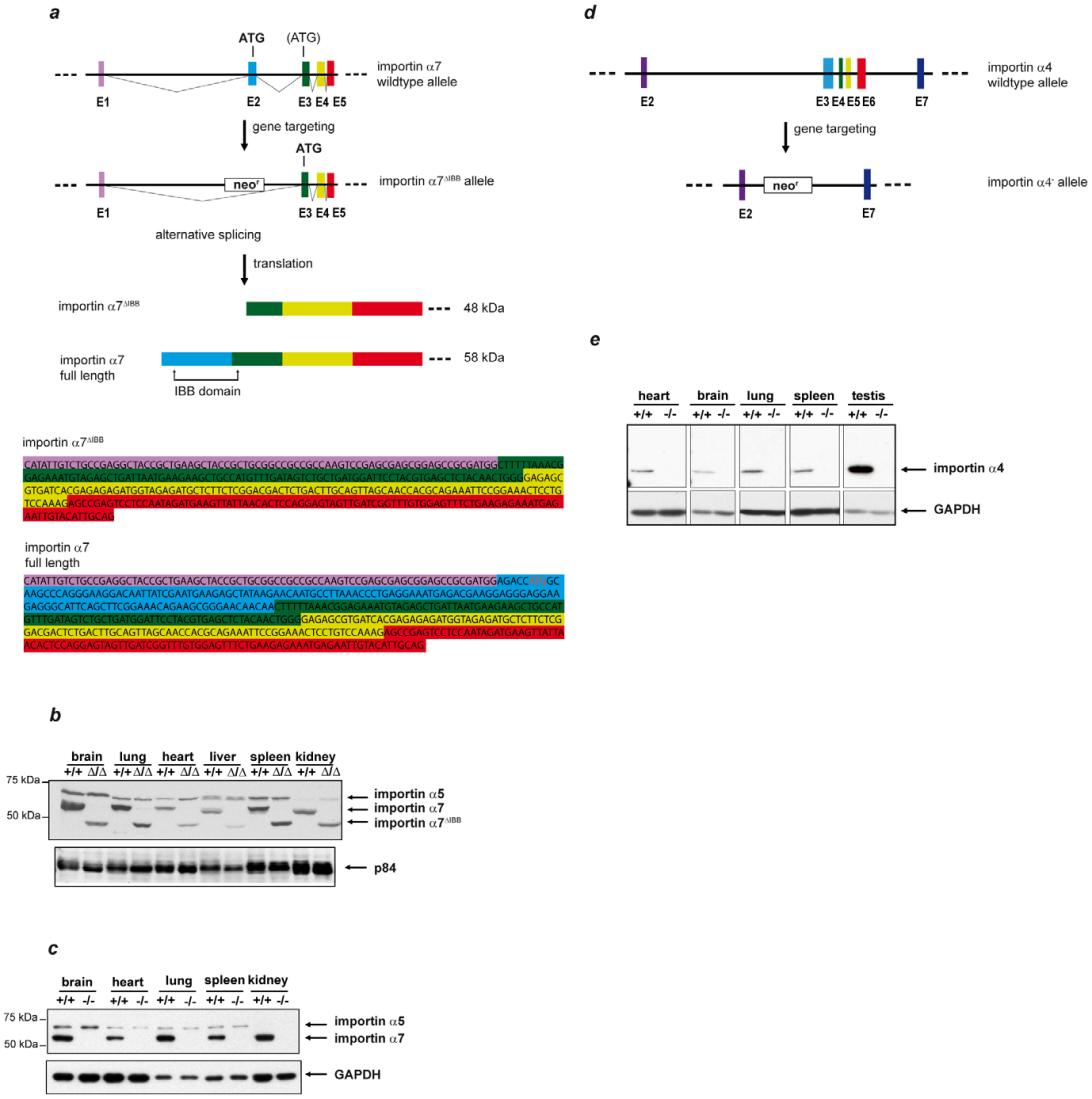
Gene targeting of importin α7 and importin α4. **a**, Gene targeting strategy for importin α7^ΔIBB/ΔIBB^ mice. After homologous recombination, exon 2 is replaced by a neomycin resistence (neo^r^) cassette with a polyadenylation site (pA). Since transcription does not always stop at pA, a splicing variant is sometimes generated, carrying an in frame translational start site in exon 3. The sequence of this variant is shown in the lower panels. **b**, Western blot analysis of different tissues with an antibody recognizing the C-terminus of importin α7 revealed the absence of the 58 kDa full length protein in α7^ΔIBB/ΔIBB^ (Δ/Δ) mice. However, a new protein is found which cannot be detected in wildtype (+/+) tissues and is about 10 kDa smaller explained by the alternative mRNA. This protein lacks the importin β binding domain (ΔIBB). The antibody shows a cross reaction with importin α5. Lower panel: An antibody against p84 was used as loading control. **c**, Western blot of importin α7^−/−^ tissues. Absence of importin α7 protein in importin α7^−/−^ tissues was confirmed by Western blot. The antibody shows a cross reaction with importin α5. Lower panel: An antibody against GAPDH was used as loading control. **d**, Gene targeting construct of importin α4^−/−^ mice. After homologous recombination, exons 3 to 6 are replaced by a neomycin resistence (neo^r^) cassette. **e**, Western blot analysis of different tissues with an antibody recognizing the N-terminus of importin α4 revealed the absence of the 58 kDa protein in α4^−/−^ (−/−) compared to wildtype (+/+) mice. An antibody against GAPDH was used as loading control.

To clarify the specific function of importin α7 in mice, we generated importin α7 knockout mice by deleting exon 2 of the gene (Fig. 1a). Unexpectedly, due to alternative splicing, these mice (α7^ΔIBB ΔIBB^) express an mRNA, which contains a cryptic translational start site in exon 3 and thus codes for an importin α7 protein lacking the importin β-binding domain (Fig. 1a, b). This domain is essential for the coupling of importin α-cargo protein complexes to importin β during nuclear import. To exclude any residual function or dominant negative effects of this shortened version of importin α7, we generated a second mouse line (α7^−/−^) containing a gene trap cassette in intron 1 of the importin α7 gene. Western blot analyses of different tissues confirmed the absence of importin α7 protein in α7^−/−^ animals (Fig. 1c). Both mouse lines (α7^ΔIBB/ΔIBB^ and α7^−/−^) were analysed in parallel. They were viable, without obvious morphological abnormalities, and histological analyses of heart, kidney, liver, lung, and spleen showed no pathological phenotypes.

### Importin α7 deficient embryos stop development at the two-cell stage

**Figure 2:**
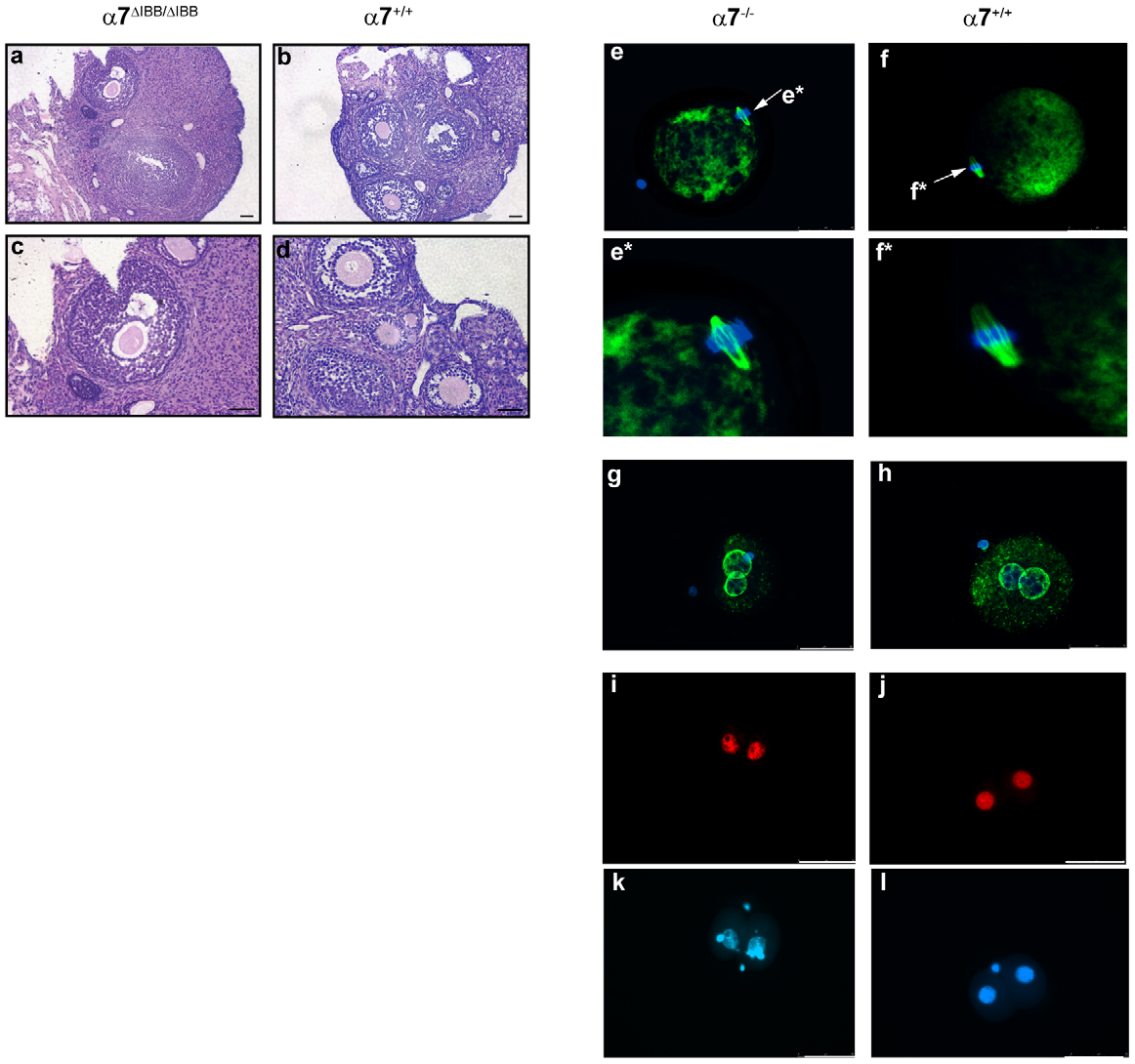
Ovarian histology, visualization of meiotic spindle, pronuclear membrane and BrdU-incorporation in importin α7-deficient embryos. **a-d**, Hematoxylin and eosin staining of ovaries of importin α7-deficient mice. Ovaries of importin α7^ΔIBB/ΔIBB^ mice (a, c) were indistinguishable from ovaries of control females (α7^+/+^, b, d). All stages of follicle development and corpora lutea were evident. **e-f**, Visualization of the meiotic spindle in importin α7-deficient oocytes. After ovulation, oocytes complete meiosis I and enter meiosis II, where they arrest at metaphase. α-tubulin staining shows a normal appearance of the meiotic spindle in importin α7-deficient oocytes (α7^−/−^, e), compared to wildtype (α7^+/+^, f). Tubulin staining was performed in three independent experiments each with 4–10 embryos. Corresponding DNA labelling with Hoechst 33258 is shown in blue, **g–h**, Nucleoporin staining in importin α7-deficient zygotes. The antibody recognizes conserved FXFG repeats of nuclear pore complex proteins. Staining of the nuclear envelope of both pronuclei (shown in green) revealed no difference between importin α7-deficient (α7^−/−^, i) and wildtype (α7^+/+^, j) zygotes. For nucleoporin staining, five independent experiments each with 4–10 embryos were carried out. Corresponding DNA labelling with Hoechst 33258 is shown in blue. **i-l**, Second round of DNA replication in importin α7-deficient embryos. Late zygotes that had already completed the first round of DNA replication, were labelled with BrdU. Immunofluorescence analysis shows BrdU incorporation in both nuclei (red) of arrested importin α7^−/−^ embryos (i) and wildtype embryos (α7^+/+^, j), indicative of entry into S phase. For BrdU labelling, five independent experiments each with 4–10 embryos were carried out. Corresponding DNA labelling with Hoechst 33258 is shown in blue (k, l). Scale bar 50 µm.

Mating of α7^ΔIBB/ΔIBB^ and α7^−/−^ female mice with wildtype males yielded no offspring, while heterozygous females of both lines were completely normal and fertile. To determine the cause of female infertility in importin α7-deficient mice, we examined ovarian histology of importin α7^ΔIBB/ΔIBB^ females. Ovaries from these animals were indistinguishable from those of control females and all stages of follicle development and corpora lutea were evident (Fig. 2a–d). Analysis of oocytes after ovulation showed regular morphology and α-tubulin staining revealed normal appearance of meiotic spindles in these cells (Fig. 2e–f).

Independent of the way of induction of ovulation (spontaneous or superovulation), zygotes with two visible pronuclei could be recovered from oviducts of α7^ΔIBB/ΔIBB^ females indicating that oocytes were able to get fertilized *in vivo.* However, when embryos developed *in vivo* were isolated at 2.5 days after fertilization, all wildtype embryos had developed to four-cell and eight-cell stages, while only 6% of recovered embryos of importin α7^ΔIBB/ΔIBB^ females had reached the two-cell stage and no single four-cell-embryo could be detected. For analysis of *in vitro* development of importin α7-deficient embryos, superovulated females were mated and zygotes were collected and cultured *in vitro*. Whereas first cleavage occurred in most of the *in vitro* cultured wildtype embryos, we observed only very few two-cell embryos of importin α7^ΔIBB/ΔIBB^ and importin α7^−/−^ females (Tab. 1). Further *in vitro* culture revealed complete arrest at the two-cell stage. Thus, a strongly reduced frequency of the first cleavage of zygotes and a complete developmental block at the two-cell stage account for infertility of females lacking importin α7.

**Table 1:** *In vitro* development of embryos.

genotype of females	number of zygotes isolated	embryos developed to		
		two-cell stage	four-cell stage	blastocyst
wildtype(n = 13)	257	204/257 (79.4%)	97/121 (80.2%)	59/83 (71.1%)
α7^ΔIBB/ΔIBB^ (n = 10)	195	75/195 (38.5%) *	0/75 (0%) *	
α7^−/−^(n = 14)	121	49/121 (40.5%) *	0/49 (0%) *	

Zygotes were isolated at multiple sessions over a period of one year and *in vitro* development was studied. The numbers of embryos from all time points were cumulated. Wildtype littermates of α7 homozygous mutant mice were used as controls. The percentages of two cell stage, four cell stage, and blastocysts are calculated relative to the number of zygotes, two- and 4-cells stages, respectively.

*p<0.0001 compared to the control group, Pearson's chi-square test.

Parthenogenetically activated oocytes of importin α7^ΔIBB/ΔIBB^ females with successful pronuclear formation showed a markedly decreased capability to develop into two-cell embryos (Tab. 2). Again no further cleavage could be observed. These results indicate that the developmental arrest in importin α7-deficient embryos is independent of paternal factors.

**Table 2:** Parthenogenetic activation of oocytes.

genotype of females	Cleavage of activated embryos to	
	two-cell stage	blastocyst
wildtype(n = 8)	154/182 (84.6%)	68/182 (37.3%)
α7^ΔIBB/ΔIBB^ (n = 8)	37/133 (27.8%) *	0/133 (0%) *

Oocytes were isolated from superovulated α7^ΔIBB/ΔIBB^ and their wildtype littermates and parthenogenetically activated. Only those oocytes that formed visible pronuclei were recorded as activated and cultured further. Percentages are calculated relative to activated oocytes.

*p<0.0001 compared to the control group, Pearson's chi-square test.

### Importin α7 is a maternal protein not involved in pronuclear membrane formation and DNA replication

In order to clarify whether anomalies in pronuclear membrane formation account for the developmental block in importin α7-deficient embryos, we performed immunocytochemistry using a specific antibody against nucleoporins. This staining revealed no abnormalities in importin α7-deficient embryos, indicating that the structure of the pronuclear membrane is not severely affected (Fig. 2g–h). Further analyses showed that both nuclei incorporated the same amount (C57Bl/6: 41.7±9.7, importin α7^−/−^: 45.0±10.9 arbitrary units) of 5-bromo-2-deoxyuridine (BrdU), suggesting that DNA replication is not disturbed in importin α7-deficient embryos (Fig. 2i-l).

**Figure 3:**
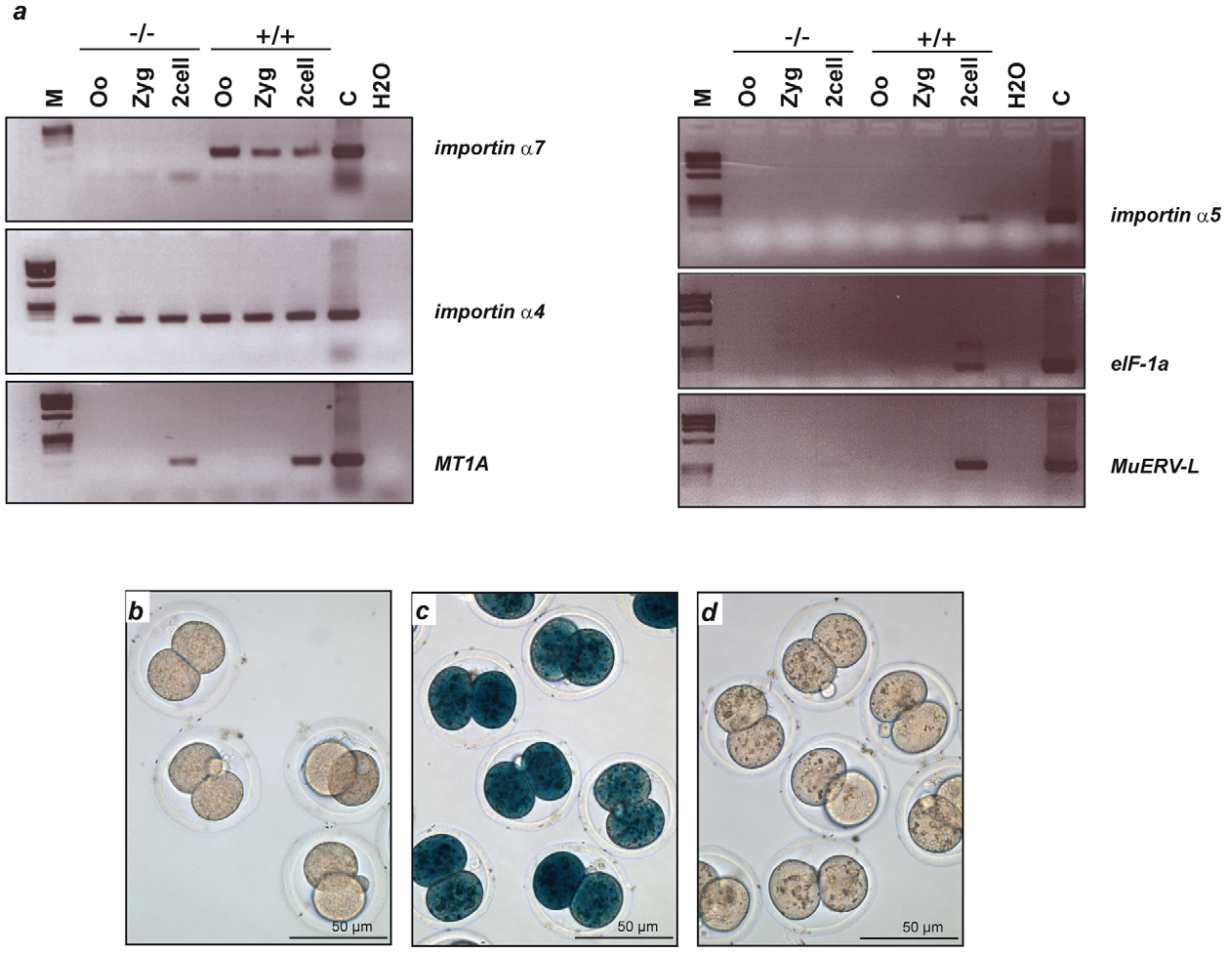
Zygotic genome activation in importin α7-deficient embryos. **a**, Expression analysis of importin α7, importin α4 and markers of ZGA in oocytes and early embryos. RT-PCR shows the maternal expression of importin α7 and α4 mRNAs in wildtype oocytes and the absence of importin α7 mRNA in embryos from importin α7^−/−^ females. Of several genes reported to be activated during ZGA only *MT1A* revealed a detectable transcript in importin α7-deficient two-cell embryos, while mRNAs for importin α5, *eIF-1a* and *MuERV-L* could not be detected, suggesting a defect in ZGA. *M* marker, *Oo* oocyte, *Zyg* zygote, *2cell* two-cell embryos, *C* control liver extract. **b–d**, β-galactosidase staining of importin α7-deficient embryos. Embryos were isolated after mating of importin α7^−/−^ females with importin α5^−/−^ males, cultivated *in vitro,* and late two-cell stages were selected for β-galactosidase staining. ZGA leads to *lacZ* expression in embryos carrying the importin α5 genetrap mutation. While wildtype embryos display a strong positive staining for β-galactosidase at the two-cell stage (c), this staining was completely absent in importin α7-deficient embryos (b) and in wildtype embryos treated with the RNA polymerase II inhibitor α-amanitin, which was used as negative control (d).

Next, we examined the presence of importin α7 mRNA in early embryos. Importin α7 mRNA could be detected in oocytes, zygotes, and two-cell embryos of wildtype mice, demonstrating maternal expression. As expected, no transcripts were found in importin α7-deficient embryos (Fig. 3a). We discovered that other members of the importin α-family, importin α4 (Kpna3, NM_008466.3), importin α2 (Kpna7), and importin α1 (Kpna2, NM_010655.3) are expressed in oocytes and early zygotes, while importin α3 (Kpna4, NM_008467.3) and α5 (Kpna1) are only detected from the two-cell stage on (Figs. 3a and Figure S1; [12], [13]). Thus, importin α1, α2, and α4 are also maternal proteins and are present in both, importin α7-deficient and wildtype embryos (Fig. 3a). Moreover, the generation of importin α4-deficient mice (Fig. 1d,e) revealed that importin α4^−/−^ females are fertile. Thus, importin α7 has unique properties in oocytes and its absence can not be compensated by other members of the family.

### Importin α7 is a maternal effect gene essential for zygotic genome activation

The developmental block of importin α7-deficient embryos coincides with the onset of ZGA. ZGA is an essential step for maternal-to-zygotic-transition and results in a novel gene expression profile which establishes the totipotent state of each blastomer in the cleavage-stage embryo [14]. First endogenous transcription in mice occurs in the late zygote stage [15] and inhibition of RNA polymerase II with α-amanitin results in a block at the two-cell stage [16]. We therefore tested the ability of importin α7-deficient embryos to activate the zygotic genome by performing reverse-transcription PCR (RT-PCR) for several genes that have recently been published to be markers of ZGA [17], [18]. mRNA expression of eukaryotic translation initiation factor 1A (*eIF-1a*), importin α5 and murine endogenous retrovirus-like gene (*MuERV-L*) was analysed in oocytes and early embryos of importin α7-deficient and control females. While wildtype two-cell embryos displayed expression of all these genes, none of these transcripts could be detected in importin α7-deficient two-cell embryos, suggesting a defect in ZGA (Fig. 3a). However, the transcript of the ZGA marker gene metallothionein 1A (*MT1A*) could be detected in two-cell stages of importin α7-deficient embryos, albeit at a markedly lower level than in wildtype.

As a second approach to analyse ZGA, we performed β-galactosidase staining of embryos from importin α7^−/−^ females after mating with importin α5^−/−^ males [19]. These mice carry a *lacZ* cassette under control of the importin α5 promoter, leading to the formation of *lacZ* mRNA when the ZGA marker gene importin α5 is transcribed. Wildtype embryos displayed strong staining for β-galactosidase in the late two-cell stage, while no staining could be detected in importin α7-deficient embryos, confirming the RT-PCR data (Fig. 3b–d). These results demonstrate that ZGA is severely disturbed in importin α7-deficient embryos.

## Discussion

In order to investigate the function of importin α7 in mice, we generated a knockout mouse line where importin α7 is completely deleted and a mouse line expressing a shortened form of importin α7 lacking the IBB domain. Homozygous females of both mouse lines are unable to produce offspring. We show that fertilized oocytes of these females display an arrest in early embryonic development. While the majority of zygotes fail to undergo first cleavage, few zygotes develop into two-cell stages, where they arrest.

Importin αparalogs has been shown not only to be involved in nuclear transport processes but also to play a role in spindle assembly and nuclear membrane formation [2]–[4]. However, analyses of the meiotic spindle in oocytes revealed normal spindle morphology. To further define the role of importin α7 in early embryonic development, we analysed formation of the pronuclear membrane which showed a regular structure in early zygotes. These findings indicate that importin α7 contributes to early embryonic development via a mechanism not involving spindle formation and nuclear membrane assembly. Using parthenogenetic activation of oocytes we also show that the developmental arrest in embryos from importin α7-deficient females is independent of paternal factors. Furthermore, we demonstrate that these embryos progress through G1 and successfully enter S phase.

We show here that importin α7 is a maternally expressed protein. Presently, fourteen maternal-effect genes are known [14], [20], [21], which by definition are expressed in the oocyte and are essential for normal embryonic development. Depletion of these genes leads to either arrest primarily at the one-cell stage (*hsf1, Npm2, Zar1, Stella*), a two-cell stage delay (*CTCF*), arrest at the two-cell stage (*Mater, brg1, mHR6a, Ago2*) or arrest at later embryonic stages (*Pms2, Dnmt3a, Dnmt1o, Ezh2, E-cadherin*). A crucial step in the maternal-to-zygotic-transition is the activation of the embryonic genome [16]. Of the known maternal-effect genes only *Mater*, *Npm2*, *Zar1* and *brg1* have been shown to display a severe disturbance of ZGA [14], [22]–[24]. Our findings demonstrate that ZGA is severely disturbed in importin α7-deficient embryos. With importin α7 we present here a new member of this group of proteins important for early embryonic development. We show that the paralogous importin α4 is also maternally expressed, but not sufficient to compensate for the lack of importin α7 and its depletion does not cause infertility, suggesting that specific functions of importin α7 are essential for early mouse development. Since importin α5 is a zygotically expressed protein, a whole subfamily of importins [7], [25] is missing in early importin α7 knockout embryos. This may explain the severity of the phenotype. Recently, a novel member of the α importin family, importin α2 (Kpna7) (Genbank entry AY950703, [13], [25]), has also been described to be maternally expressed and it was shown that its depletion leads to a comparable phenotype as the importin α7 knockout, a stop in development at the two-cell stage [12]. However, in this case the phenotype is not completely penetrant and half of the embryos reach the blastocyst stage. This maybe due to the fact that importin α2 belongs to the same subfamily of α importins as importin α1 [25], which is also maternally expressed and may therefore partially compensate for the lack of importin α2. Taken together, α importins play important but yet undefined roles in early preimplantation development of mammals.

## Materials and Methods

### Superovulation and embryo culture

Local German authorities (Landesamt für Gesundheit und Soziales, Berlin) approved the animal studies with standards corresponding to those prescribed by the American Physiological Society (Approval number: G0180/05). Superovulation and embryo culture was performed as described elsewhere [26]. Briefly, female mice were injected i.p. with 5 IU Intergonan (Intervet) followed by injection of 5 IU Ovogest (Intervet) within 48–50 h. To isolate zygotes, mice were mated with C57Bl/6 males and checked for the presence of a copulatory plug on the following morning. Animals were sacrificed and oviducts were transferred to M2 medium (Sigma) containing hyaluronidase (0.1% w/v; Sigma). After release from the oviduct oocytes and embryos were cultured *in vitro* in M16 medium (Sigma) at 37 °C under 5% CO_2._

### Parthenogenetic activation of oocytes

Parthenogenetic activation of oocytes was performed as described elsewhere [27]. Briefly, oocytes were isolated from superovulated females 14–16 h after Ovogest injection and incubated for 1 hour in Ca^2+^ and Mg^2+^ -free M16 medium containing 2 mM Sr^2+^ at 37 °C under 5% CO_2._ To obtain diploid parthenogenetic embryos, the oocytes were cultured 7–8 hours in the presence of 5 µg/ml cytochalasin B (Sigma). Efficiency of pronuclear formation was analyzed 10–12 h after treatment. Oocytes were observed under an inverted microscope with Nomarski optics (Zeiss). Those oocytes that formed visible pronuclei were recorded as activated and cultivated further.

### Western Blot

The generation of the anti-importin α7 antibody is described elsewhere [7]. This antibody was raised against the C-terminus of human importin α7 (peptide PEAPMEGFQL), which is identical to the murine importin α7 protein. Due to high sequence homology at the C-terminus of importin α7 and importin α5 (9 out of 10 aminoacids are identical), anti-importin α7 antibody shows a cross reactivity to importin α5 which appears as a distinct band in Western blots. The anti-importin α4 antibody was raised in rabbits against the peptide sequence MAENPGLENHRIC of the murine importin α4 protein using standard protocols [7].

12 µg tissue extract were loaded on a 10% SDS gel. After transfer of proteins, the PVDF membrane was blocked by Tris-buffered saline/0.1% Tween (TBST) with 5% skim milk powder and subsequently incubated with primary anti-importin α7 antibody (diluted 1∶40,000 in TBST with 1% skim milk powder) or anti-importin α4 antibody (diluted 1: 20,000 in TBST with 1% skim milk powder) at 4 °C over night. On the next day, the membrane was incubated with secondary antibody against rabbit IgG conjugated with horseradish peroxidase (1∶2,000; Pierce) for 1 h at room temperature and detection was performed using the ECL Super Signal West Dura reagent (Pierce). For loading control, staining of the Western blot membranes was performed with Ponceau S solution (Sigma Aldrich) according to the manufacturer's instructions.

### RNA isolation and RT-PCR

Total RNA was isolated from unfertilized oocytes and embryos using the RNeasy Mini Kit (QIAGEN) and incubated in a 20 µl reaction mixture containing 100 U of Super Script II Reverse Transcriptase (Invitrogen) and 200 ng of random primer (Roche) at 25 °C for 10 minutes, followed by 30 minutes incubation at 42 °C and inactivation for 15 minutes at 70 °C. The cDNA was diluted to 0.5 embryos equivalent/µl. For PCR, a 10 µl reaction mixture consisted of 1 µl of the cDNA solution, 10 ng of each primer, 2 µM dNTP, 20 mM MgCl_2_, and 0.2 U/µl Taq DNA polymerase (Invitrogen). For detailed information on primer sequences and PCR conditions see Table S1.

### β-galactosidase staining of embryos

Cultured two-cell embryos were rinsed in phosphate buffer, fixed for 5 minutes in fixation solution, washed 3 times for 5 minutes in wash buffer and incubated with X-gal stain overnight at 37 °C (for detailed protocol see http://www.med.umich.edu/tamc/laczstain.html). For control, wildtype embryos were treated with α-amanitin (Sigma, 24 µg/ml) over night prior to fixation. After staining, embryos were placed in wash buffer und stored at 4 °C. For visualization, a Leica DMI6000B microscope (Leica) with a Leica DFC 420 camera (Leica) was used.

### Generation of α7^ΔIBB/ΔIBB^, α7^−/−^, and α4^−/−^ mice

To generate the α7^ΔIBB^ targeting construct, a 1300 bp-long sequence of intron 1 and 5000 bp-long sequence downstream of exon 2 of the importin α7 gene were cloned in a targeting vector [28]. After homologous recombination in embryonic stem (ES) cells, exon 2, which bears the translational start site for the importin α7 protein, was deleted. ES cell manipulation was performed as described previously [28]. Briefly, ES cells were electroporated with the linearised construct and after a double selection process with neomycin and gancyclovir, 176 clones were picked. We identified 5 positive clones by PCR of which one was chosen for blastocyst injection.

For the generation of α7^−/−^ mice, ES cells with a gene trap mutation in the importin α7 gene (clone AJ0609) were purchased form Sanger Gene Trap Resource and directly used for blastocyst injection.

The knockout construct for importin α4^−/−^ was cloned in a targeting vector [28] using a 1100 bp-long sequence of intron 2 and a 5000 bp-long sequence downstream of exon 6 of the importin α4 gene. After homologous recombination in ES cells exons 3–6 are deleted. ES cells were electroporated with the linearised vector and after a double selection process with neomycin and gancyclovir, 136 clones were obtained. Two positive clones were identified by PCR and injected into blastocysts.

From all injected ES cell clones we obtained germline chimeras, which were subsequently bred with C57Bl/6 mice. Both colonies of α7-mutant mice were maintained by breeding the resulting heterozygous mice. Importin α4-deficient mice were backcrossed to C57Bl/6 genetic background, bred to the homozygosity and, since they were fertile, maintained as −/− colony. For genotyping of importin α7^−/−^ mice, we performed RT-PCR with RNA isolated from tail biopsies. Genotyping of importin α7^ΔIBB/ΔIBB^ and α4^−/−^ mice was performed using PCR on genomic DNA. The primer sequences and PCR conditions are listed in Table S1.

### Hematoxylin and eosin staining of ovaries

Ovaries from 16 week old mice were isolated and paraffin embedded. Slides with sections were deparaffinized and rehydrated by incubation in descending ethanol series. Slides were incubated for 3 minutes in hematoxylin, subsequently rinsed with water for 20 minutes and placed in eosin for 6 minutes. After dehydration by ascending ethanol series, slides were mounted in Eukitt (Kindler). For visualization, a Leica DMI6000B microscope (Leica) with a Leica DFC 420 camera (Leica) was used.

### BrdU labelling of embryos

After isolation embryos were incubated in M16 medium until 24 hours post Ovogest injection to get late zygotes in which the first round of replication is completed. Zygotes were transferred to M16 medium supplemented with BrdU (Sigma) to a final concentration of 50 µM and incubated overnight. At the end of the incubation period, two-cell embryos were rinsed in PBS, fixed in 3.7% paraformaldehyde (PFA) in PBS at room temperature for 15 minutes and permeabilised in 0.1% Triton X-100 in PBS/1% normal donkey serum (NDS) for 30 minutes. After washing in PBS/0.05% Tween/0.1% NDS, embryos were placed in 2 M HCl/PBS for 10 minutes to denature the DNA. Embryos were then extensively washed in PBS/0.05% Tween/0.1% NDS and blocked with PBS/0.05% Tween/5% NDS for 1 h at room temperature. Detection of BrdU-DNA was performed using a monoclonal anti-BrdU antibody (Boehringer; 1∶50 diluted, incubation overnight at 4 °C) and a secondary anti-mouse IgG antibody coupled to Cy3 (Dianova; 1∶2,000 diluted, incubation for 1 hour at room temperature). Embryos were incubated with 5 µg/ml Hoechst 33258 (Hoechst) for 15 minutes and mounted with Fluorescence Mounting Medium (DAKO). BrDU staining was quantified using the Image J program.

### Immunofluorescence staining of oocytes and embryos

For visualization of the meiotic spindle, oocytes isolated from oviducts of superovulated mice were collected, washed in PBS and fixed in 3.7% PFA in PBS at room temperature for 15 minutes. After permeabilisation in PBS/0.25% Tween for 5 minutes at room temperature, oocytes were transferred to blocking buffer (containing PBS/2% bovine serum albumine (BSA)/2% normal goat serum/0.1 M glycine/0.01% Triton X-100) and incubated at 4° overnight. Then, oocytes were incubated with a FITC conjugated anti-α-tubulin antibody (Sigma; 1∶100 in PBS/0.1% BSA) for 2 h at 37 °C. At the end of the incubation period, oocytes were washed in PBS/0.1% BSA, DNA was counterstained with 5 µg/ml Hoechst 33258 for 15 minutes and mounted with Fluorescence Mounting Medium.

For visualisation of nuclear pores, zygotes isolated from oviducts of superovulated and mated mice were collected, washed in PBS and fixed in 3.7% PFA in PBS at room temperature for 15 minutes. After washing, zygotes were permeabilised for 20 minutes in PBS/0.5% Triton X-100/0.1% BSA, transferred to blocking buffer and incubated at 4 °C overnight. Incubation with anti-nucleoporin antibody (MAb414, Covance; 1∶100 diluted) was performed for 2 h at 37 °C. At the end of the incubation period, oocytes were washed in PBS/0.1% BSA and incubated with Cy2-conjugated donkey anti-mouse IgG antibody (Dianova; 1∶500 diluted). After washing DNA was counterstained with 5 µg/ml Hoechst 33258 for 15 minutes and mounted with Fluorescence Mounting Medium. Immunofluorescence was detected using the Leica DMI 6000B fluorescence microscope with a Leica DFC 350FX fluorescence camera (Leica).

### Statistical analyses

Statistical analysis was performed using Pearson's chi-square test.

## Supporting Information

Figure S1 Expression analysis of importin α1, α2, and α3 in oocytes and early embryos. RT-PCR shows the maternal expression of importin α1 and α2 mRNAs in wildtype oocytes and the zygotic activation of importin α3. The absence of importin α7 mRNA in embryos from importin α7^−/−^ females does not interfere with the expression of the other α importins. The sequence of importin α2 (Kpna7) was identified by homology of sequence tags from GenBank database to other α-importins and subsequent sequencing of the respective cDNA clones. The sequence of one complete clone was deposited as entry AY950703.
(JPG)

Table S1 Primer sequences and conditions for PCR.
(DOC)
