# Supplementary figures and images for: Importin α7 Is Essential for Zygotic Genome Activation and Early Mouse Development
Source: PLoS One. 2011 Mar 29;6(3):e18310. doi: 10.1371/journal.pone.0018310 (PMC3066239; doi:10.1371/journal.pone.0018310)

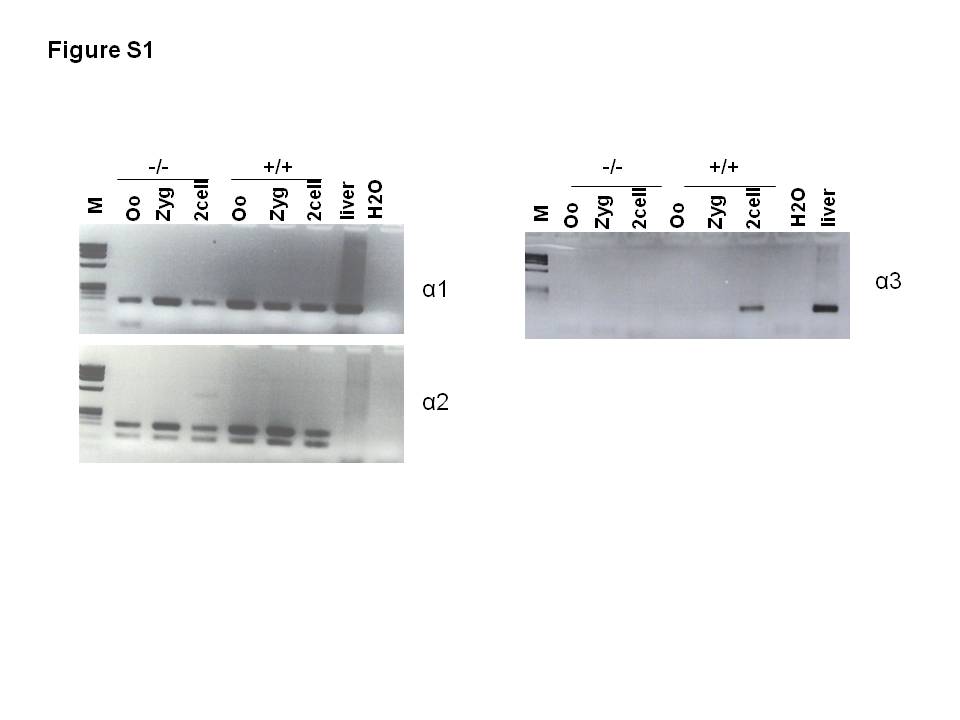

Supplement: Figure S1 — Expression analysis of importin α1, α2, and α3 in oocytes and early embryos. RT-PCR shows the maternal expression of importin α1 and α2 mRNAs in wildtype oocytes and the zygotic activation of importin α3. The absence of importin α7 mRNA in embryos from importin α7−/− females does not interfere with the expression of the other α importins. The sequence of importin α2 (Kpna7) was identified by homology of sequence tags from GenBank database to other α-importins and subsequent sequencing of the respective cDNA clones. The sequence of one complete clone was deposited as entry AY950703. (JPG) [file pone.0018310.s001.jpg]
