# Supplementary material for: Importin α7 Is Essential for Zygotic Genome Activation and Early Mouse Development
Source: PLoS One. 2011 Mar 29;6(3):e18310. doi: 10.1371/journal.pone.0018310 (PMC3066239; doi:10.1371/journal.pone.0018310)
Supplement: Table S1 — Primer sequences and conditions for PCR. (DOC) [file pone.0018310.s002.doc]

**Supporting Information**

**Table S1** Primer sequences and conditions for PCR.

| name | primer | | primer sequence | annealing temperature | number of cycles |
| --- | --- | --- | --- | --- | --- |
| MuERV-L | sense  antisense | | 5’-TTTCTCAAGGCCCACCAATAGT-3’  5’-GACACCTTTTTTAACTATGCGAGCT-3’ | 62°C | 30 |
| MT1A | sense  antisense | | 5’-CACCAGATCTCGGAATGGAC-3’  5’-AGCAGCTCTTCTTGCAGGAG-3’ | 58°C | 35 |
| eIF-1a | sense  antisense | | 5’-TTTGGTCACTACTCAGGAGG-3’  5’-ATCAGAAGCAACTGGGACAC-3’ | 60°C | 35 |
| importin α1 | sense  antisense | | 5’-GTCACCGGGAAATTTAAATCG-3’  5’-TGCAGGAACAAGGAGGAAAG-3’ | 60°C | 35 |
| importin α2 | sense  antisense | | 5’-TCAGCCCCTGATTGAACTCC-3’  5’-GGCATTATTGGAGATGACACAG-3’ | 60°C | 35 |
| importin α3 | sense  antisense | | 5’-GAACAAAGGCCGTGACTTGG-3’  5’-AATCACCATCTATATCGGAGTC-3’ | 62°C | 30 |
| importin α4 | sense  antisense | | 5’-TGACAGTTGAACTCCGGAAG-3’  5’-AGCACTCAACTGGACTACAG-3’ | 60°C | 35 |
| importin α5 | sense  antisense | | 5’-CGTCTCCCTCTTCGTAGTG-3’  5’-CTTCTTCCTCCCTCCTCCTG-3’ | 58°C | 35 |
| importin α7 | sense  antisense | | 5’-AGGCTACCGCTGAAGCTACC’-3’  5’-CATTTCCTCAGGGTTTAAGGC-3’ | 58°C | 35 |
| Genotyping primers | | |  |  |  |
| **importin α7^ΔIBB^**  wildtype allele  mutant allele | | sense  antisense  sense  antisense | 5'-GCTCTTAAGCCAGAGGGAAC-3'  5'-CATTTCCTCAGGGTTTAAGGC-3'  5’-AGGATCATCAACATTGAAGGTA-3’  5’-GTTGTGCCCAGTCATAGCCGAATAGCC-3’ | 58°C  55°C | 35  30 |
| **importin α7^-^**  wildtype allele*  mutant allele | | sense  antisense  sense  antisense | 5’-CATATTGTCTGCCGAGGCTAC-3’  5’-ACCGATCAACTACTCCTGGAG-3’  5’-GAACAAGATGGATTGCACGCAG-3’  5’-CCAAGCTCTTCAGCAATATCACG-3’ | 60°C  61°C | 35  35 |
| **importin α4^-^**  wildtype allele  mutant allele | | sense  antisense  sense  antisense | 5’-GAAGATTCAGATGTTGATGGTG-3’  5’-ACTAGAATTGGTAAAATCCCAG-3’  5’-GTAGTATATTTTGTCAGTTGG-3’  5’-GTTGTGCCCAGTCATAGCCGAATAGCC -3’ | 58°C  60°C | 35  30 |
